# Effects of Cathepsin S Inhibition in the Age-Related Dry Eye Phenotype

**DOI:** 10.1167/iovs.64.11.7

**Published:** 2023-08-04

**Authors:** Jeremias G. Galletti, Kaitlin K. Scholand, Claudia M. Trujillo-Vargas, Wolfgang Haap, Tiago Santos-Ferreira, Christoph Ullmer, Zhiyuan Yu, Cintia S. de Paiva

**Affiliations:** 1 Department of Ophthalmology, Baylor College of Medicine, Houston, Texas, United States; 2 Institute of Experimental Medicine, Buenos Aires, Argentina; 3 Department of Biosciences, Rice University, Houston, Texas, United States; 4 Grupo de Inmunodeficiencias Primarias, Facultad de Medicina, Universidad de Antioquia, UdeA, Medellín, Colombia; 5 Roche Pharma Research and Early Development, F. Hoffmann-La Roche Ltd, Basel, Switzerland

**Keywords:** aging, lacrimal gland, dry eye

## Abstract

**Purpose:**

Aged C57BL/6J (B6) mice have increased levels of cathepsin S, and aged cathepsin S (*Ctss^−^^/^^−^*) knockout mice are resistant to age-related dry eye. This study investigated the effects of cathepsin S inhibition on age-related dry eye disease.

**Methods:**

Female B6 mice aged 15.5 to 17 months were randomized to receive a medicated diet formulated by mixing the RO5461111 cathepsin S inhibitor or a standard diet for at least 12 weeks. Cornea mechanosensitivity was measured with a Cochet–Bonnet esthesiometer. Ocular draining lymph nodes and lacrimal glands (LGs) were excised and prepared for histology or assayed by flow cytometry to quantify infiltrating immune cells. The inflammatory foci (>50 cells) were counted under a 10× microscope lens and quantified using the focus score. Goblet cell density was investigated in periodic acid–Schiff stained sections. *Ctss^−^^/^^−^* mice were compared to age-matched wild-type mice.

**Results:**

Aged mice subjected to cathepsin S inhibition or *Ctss^−^^/^^−^* mice showed improved conjunctival goblet cell density and cornea mechanosensitivity. There was no change in total LG focus score in the diet or *Ctss^−^^/^^−^* mice, but there was a lower frequency of CD4^+^IFN-γ^+^ cell infiltration in the LGs. Furthermore, aged *Ctss^−^^/^^−^* LGs had an increase in T central memory, higher numbers of CD19^+^B220^−^, and fewer CD19^+^B220^+^ cells than wild-type LGs.

**Conclusions:**

Our results indicate that therapies aimed at decreasing cathepsin S can ameliorate age-related dry eye disease with a highly beneficial impact on the ocular surface. Further studies are needed to investigate the role of cathepsin S during aging.

The ocular surface is a complex and dynamic system constantly in contact with the external environment. It is subject to various challenges that can lead to inflammation, infection, and other diseases. To maintain the health and function of the ocular surface, a sophisticated and finely regulated immune system is in place to detect and respond to any threats.^1^^,^^2^ Conversely, immune dysregulation is a core pathophysiologic mechanism in several ocular surface disorders. Although not a disease itself, aging also brings about dysregulatory changes in the ocular surface and is thus associated with an increased prevalence of dry eye and allergy.^3^

Among the numerous age-related changes described, aged ocular surface antigen-presenting cells (APCs) are more mature and better promote the differentiation of proinflammatory Th1 CD4^+^ T cells than their young counterparts.^4^ In line with this, we reported that aged mice display impaired ocular mucosal tolerance,^1^ a key regulatory immune mechanism to keep inflammation at bay.^3^^,^^5^^–^^10^ APCs are master regulators of the immune response because they prime naive T cells into different Th subsets. Antigen presentation involves the processing of antigen-derived peptides by the concerted action of lysosomal proteases. Many lysosomal enzymes that process major histocompatibility complex II (MHC II)–associated peptides have been described.^11^^–^^14^ Although most of these proteases have rather broad cleavage specificities, the system is not entirely redundant. Age-related changes in antigen presentation-associated proteases are thought to contribute to the increase in autoimmunity observed in the elderly.^15^

Cathepsin S is a cysteine protease primarily expressed in the lysosomes of phagocytic cells, such as macrophages, APCs, and lacrimal gland acinar cells.^16^^,^^17^ One of its primary functions is degrading the MHC II–associated invariant chain (Ii). The Ii peptide acts as a placeholder within the antigenic peptide groove of the MHC II complex during its assembly in the endoplasmic reticulum to prevent the unwanted presentation of other endogenous peptides as potential antigens. During a later phase of the antigen presentation process that takes place within the lysosomes, cathepsin S cleaves the li peptide in the MHC II groove into the CLIP peptide (class II invariant chain-associated peptide), which can then be exchanged for other antigenic peptides with higher affinity for the MHC II complex.^11^^–^^13^^,^^18^ Thus, cathepsin S plays a crucial role in MHC class II–mediated antigen presentation, and conversely, higher levels of cathepsin S have been linked to the generation of autoreactive CD4^+^ T cells due to increased MHC II presentation time.^18^^–^^20^ In addition, a transgenic mouse with overexpression of cathepsin S displays spontaneous age-related systemic inflammation.^21^ Elevated cathepsin S activity in tears has been proposed as a potential biomarker for dry eye and Sjögren syndrome, as published data have shown increased activity in non–Sjögren syndrome dry eye patients compared to healthy controls.^22^

Aging is a multifactorial complex biological process accompanied by inflammation and immune infiltration in many tissues, including the eye and lacrimal gland.^23^^–^^25^ On the other hand, increased levels of cathepsin S in plasma correlate with higher mortality in the elderly.^26^^,^^27^ The age-associated increase in inflammation might create a vicious cycle, as inflammatory cytokines like IFN-γ, TNF, and IL-1β are known to upregulate cathepsin S,^28^^,^^29^ which in turn upregulates IL-1β and TNF (TNF is no longer named TNF-α^30^). In line with this, cathepsin S increases with aging in the retina, brain, and lacrimal gland of mice,^17^^,^^31^^,^^32^ and we have previously shown that aged tears have high levels of active cathepsin S.^33^

Cathepsin S inhibitors have been developed and tested in many preclinical models of autoimmunity to decrease the generation of autoreactive T cells by interfering with MHC II presentation.^19^^,^^34^^–^^36^ Because Sjögren syndrome and aged lacrimal glands share many similarities and activation of pathways related to immune activation,^10^ and because our work showed that 1-year-old *Ctss*^−/−^ mice have less severe dry eye phenotype than aged wild-type controls,^33^ we hypothesized that a diet containing a cathepsin S inhibitor would also be beneficial during aging. The cathepsin S inhibitor RO5461111 was chosen since it is a selective inhibitor of cathepsin S.^34^

The objective of this study was twofold: (1) to investigate the effects of RO5461111 on age-related dry eye phenotype and (2) to investigate the effects of pharmacologic and genetic deletion of cathepsin S on the immune infiltrates that accompany aging in the lacrimal gland. To this end, we subjected 15- to 17-month-old C57BL/6 mice to a 12- or 28-week regimen with a cathepsin S inhibitor in the diet. We observed several positive effects on the ocular surface (improvement of goblet cell density and mechanical sensitivity). While the total immune infiltrates in the lacrimal gland did not change, we observed a decrease in Th1 cells infiltrating the lacrimal glands and a change in the B-cell landscape. These results were recapitulated in aged *Ctss*^−/−^ mice. These results indicate that cathepsin S inhibition improves age-related dry eye without affecting the total immune infiltration in the lacrimal gland. Further studies are necessary to investigate if topical administration of a cathepsin S inhibitor would also benefit age-related dry eye.

## Methods

### Animals

The Institutional Animal Care and Use Committee at Baylor College of Medicine and Jackson Laboratories approved all animal experiments. In addition, all studies adhered to the Association for Research in Vision and Ophthalmology for the Use of Animals in Ophthalmic and Vision Research and the NIH Guide for the Care and Use of Laboratory Animals.^37^ The experiments were performed at the Ocular Surface Center, Department of Ophthalmology, Baylor College of Medicine (Houston, TX, USA).

C57BL/6J (B6) animals were purchased from the Jackson Laboratories (Bar Harbor, ME, USA), aged in-house, or received from the National Institute of Aging. Breeder pairs of cathepsin S knockout (*Ctss*^−/−^ mice, on a B6 background) were obtained from Dr. Thomas Reinheckel (Institute for Molecular Medicine and Cell Research University Medical Center Albert-Ludwigs, University Freiburg, Freiburg, Germany), and a breeding colony was established. For aging-related phenotype studies, B6 mice were used at 2 to 3 months (*n* = 57), 12 to 14 months (*n* = 21), 18 months (*n* = 8), and 24 months or older (*n* = 51). *Ctss*^−/−^ mice were used at 2 to 3 months (*n* = 47), 12 to 13 months (*n* = 19), 18 months (*n* = 8), and 24 months or older (*n* = 53). Additional B6 mice aged 15.5 to 17 months (*n* = 104) were subjected to a diet containing cathepsin S inhibitor, as described below.

Mice were housed in specific pathogen–free facilities of Baylor College of Medicine and Jackson Laboratories. They were kept on diurnal cycles of 12 hours/light and 12 hours/dark with ad libitum access to food, water, and environmental enrichment. No intervention was made to the mice; therefore, our experiments did not induce pain, suffering, or distress. Criteria for early euthanasia included loss of 20% or more of body weight, extensive ulcerative dermatitis, or cornea opacification. Mice subjected to early euthanasia were not included in the study.

Because dry eye is more frequent in women,^38^^,^^39^ and aged male mice do not develop corneal barrier disruption,^40^ we used female mice. An effort was made to collect multiple tissues from each mouse. A final sample size per endpoint can be found in figure legends.

### Diet Regimen

Three different cohorts of animals aged 15.5 to 17 months were randomized to receive either a medicated diet formulated by mixing the cathepsin S inhibitor (R05461111, 262.5 mg/kg chow; Roche, Switzerland)^19^^,^^34^ or a standard diet (placebo). The medicated diet was commercially prepared by LabDiet (San Antonio, TX, USA). Mice were weighed biweekly.

Cohorts 1 and 2 received a medicated diet for 12 weeks (*n* = 15/group and *n* = 21), while cohort 3 received the diet for 28 weeks (*n* = 16/group). The age at euthanasia for cohorts 1/2 and 3 was ∼17 to 19 months and 19 to 22 months, respectively. All endpoints were tested at euthanasia, but corneal sensitivity measurements were performed in live animals 1 week before euthanasia. A final sample size per endpoint can be found in the figure legends.

### Corneal Mechanical Sensitivity

Corneal sensitivity was measured under a surgical loupe with the Luneau Cochet–Bonnet instrument (Western Ophthalmics, Lynnwood, WA, USA). This instrument relies on increasing pressure as the filament shortens (range, 6–0.5 cm). This test was performed by two experimenters. While holding the animal (experimenter 1), a nylon filament was applied to the central cornea by the second experimenter. Experimenter 2 was blinded to the animal group/treatment. As the initial test, the nylon thread was extended to 4. The step technique was used; if a specific length exhibited no response, the next lower step (meaning higher pressure) was tested until a positive response was obtained. A similar technique using a higher step (meaning lower pressure) was used if the initial length tested negative. A positive response was indicated by a clear stimulus-evoked blink and retraction of the eye into the ocular orbit. The central cornea was tested six times at each filament length. The response was considered negative when the monofilament touch elicited no blink. A positive response was considered when the animal blinked more than or equal to 50% of the times tested.

### Histology, Periodic Acid–Schiff Staining, and Quantification of Focus Score

Eyes and ocular adnexa were excised, fixed in 10% formalin, paraffin embedded, and cut into 5-µm sections using a microtome (Microm HM 340E; Thermo Fisher Scientific, Waltham, MA, USA). Eye sections cut from paraffin-embedded globes were stained with periodic acid–Schiff (PAS) reagent. The goblet cell density was measured in the superior and inferior bulbar and tarsal conjunctiva using NIS-Elements software (AR, version 5.20.2; Nikon Melville, NY, USA) and expressed as the number of positive cells per millimeter.^41^

Lymphocytic infiltration foci were counted in hematoxylin and eosin–stained lacrimal gland sections by standard light microscopy using a 10× objective (Eclipse E400; Nikon) by two masked observers. A minimum of 50 mononuclear cells was counted as one focus, and the total number of foci per gland was recorded. Slides were scanned to obtain digital images using PathScan Enabler V (Meyer Instruments, Houston, TX, USA) and were calibrated according to the manufacturer's instructions (2.54 µm/px) using NIS Elements software. The lacrimal glands' total area was measured using the “autodetect area” function of the Nikon Elements software or was manually circumscribed using the polyline function. Finally, focus scores were calculated by dividing the number of foci per mm^2^ and quantifying the number of inflammatory cell foci per 4 mm^2^ tissue area.

### Flow Cytometry Analysis

Single-cell suspensions of the lacrimal glands, conjunctiva, and cervical lymph nodes were prepared as previously reported.^23^ Then, 1 × 10^6^ cells from the single-cell suspensions of the lacrimal gland and cervical lymph nodes (*n* = 7 to 10/group) were plated and then stained with different panels. Cells were blocked with CD16/CD32 (BioLegend, San Diego, CA, USA), washed, incubated with live/dead cell discriminator (IR; Invitrogen-Molecular Probes, Thermo Fisher Scientific, Waltham, MA, USA), and stained using different panels as below.

For germinal center evaluation, cells were stained with CD45 (BV510, clone 30F11, cat. #103138; BioLegend), B220 (clone RA3-6B2, cat. #553092; BD Biosciences, Franklin Lakes, NJ, USA), GL7 (PERCP CY5.5, cat. #144610; BioLegend), and CD95 (PE, cat. #152608; BioLegend). The following gating strategy was used: live CD45^+^ cells were gated after excluding live/dead dye and two sequential single-cell gates. B220^+^ cells were plotted versus side scatter area and further gated into GL7^+^ and CD95^+^ cells. The presence of autofluorescence (attributed to an age-related increase in lipofuscin)^42^ is more evident in some wavelengths than others. To circumvent this, we gated CD95^+^GL7^+^ cells based on fluorescence minus control from lacrimal gland cell suspensions.

To investigate the frequency of T follicular helper cells, we used the following antibodies: CD45 (BV510, clone 30F11; BioLegend), CD4 (FITC, clone RM4-5, cat. #11-0042-86; Invitrogen/ThermoFisher), CXCR5 (PE, clone L138D7, cat. #145504; BioLegend), PD-1 (BV421, clone 29F.1A12, cat. #135218; BioLegend), BCL-6 (PE_CY7, clone 7D1, cat. #358512; BioLegend), and IL-21 (APC, clone FFA21, cat. #17-7211-82; ThermoFisher). For this panel, single-cell suspensions were incubated with PMA and ionomycin for 5 hours.^43^ The following gating strategy was used: live CD45^+^ cells were gated after the exclusion of live/dead dye and two sequential single-cell gates. CD4^+^ were plotted and identified. CXCR5 was plotted versus PD-1, and CXCR5^+^PD-1^+^ cells were further examined based on the expression of BCL-6 and IL-21.

The following panel examined the frequency of marginal zone–like B, B follicular, and transitional B cells: CD45 (BV510, clone 30F11), B220 (clone RA3-6B2), CD93 (PECY7, clone AA41, cat. #136506; BioLegend), IgM (FITC, clone RMM-1, cat. #406506; BioLegend), and CD23 (BV421, clone B3B4, cat. #101621; BioLegend). Cells were washed and then kept on ice until data acquisition. The following gating strategy was used: live CD45^+^ cells were gated after excluding live/dead dye and two sequential single-cell gates. B220^+^ cells were plotted versus side scatter area and further gated as CD93^+^ and CD93^−^ cells. Cell populations were then plotted as IgM versus CD23, and gates were made based on fluorescence minus 1 controls. A separate experiment verified the frequency of CD19 and B220 by using the following antibodies: CD45 and B220 (as above) and CD19 (APC, clone 6D5, cat. #115512; BioLegend).

A BD LSRII Benchtop cytometer was used for data acquisition, and data were analyzed using BD Diva Software (BD Pharmingen, Franklin Lakes, NJ, USA) and FlowJo software (version 10.1; Tree Star, Inc., Ashland, OR, USA). Biological replicates were averaged.

### Statistical Analysis

Based on pilot studies, the sample size was calculated with StatMate2 Software (GraphPad Software, San Diego, CA, USA) to have at least 90% power to detect differences with an α of 0.5. Statistical analyses were performed with GraphPad Prism software (version 9.2; GraphPad Software). Data were first evaluated for normality with the Kolmogorov–Smirnov normality test. Appropriate parametric (*t*-test) or nonparametric (Mann–Whitney) statistical tests were used to compare the two age groups. Whenever adequate, one-way or two-way ANOVA or Kruskal–Wallis followed by post hoc tests were used. The final sample per experiment is shown in the figure legends.

## Results

### Protective Effects of Cathepsin S Inhibition on the Ocular Surface During Aging

**Figure 1.**
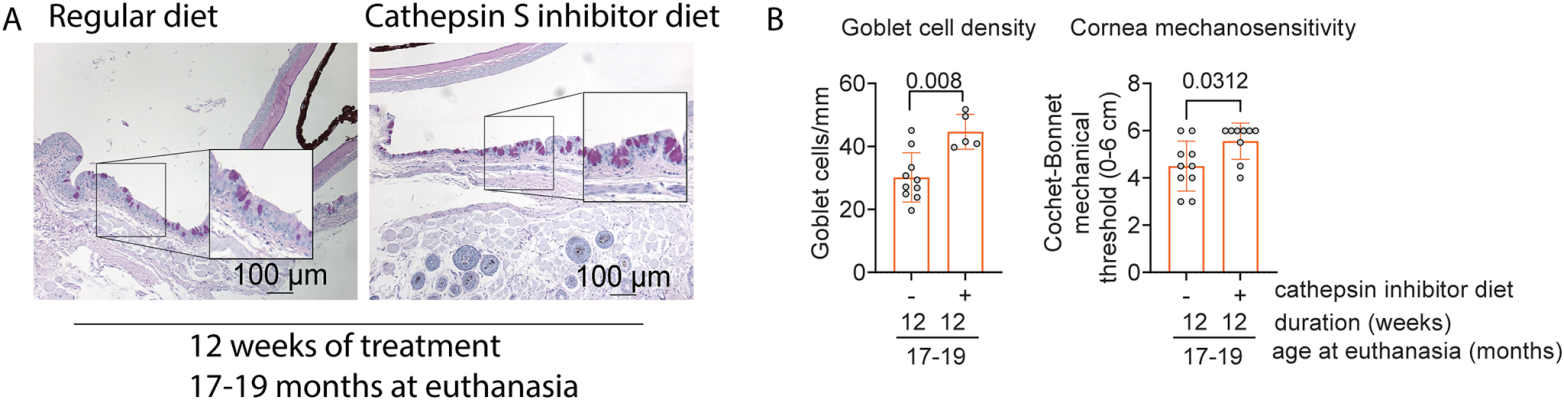
Cathepsin S inhibition diet for 12 weeks improves goblet cell density and cornea mechanosensitivity in 17- to 19-month-old mice. (**A**) Representative images of conjunctival sections stained with PAS (*purple magenta*) showing increased goblet cell density with the cathepsin S inhibition diet. *Insets* are a higher magnification of the area on the left. *Scale bar*: 100 µm. (**B**) Cumulative data of conjunctival goblet cell density after treatment. Mann–Whitney *U* test; each *dot* represents one animal, *n* = 5–10/group. (**C**) Cornea sensitivity was assessed using the Cochet–Bonnet aesthesiometer. Mann–Whitney U test, each dot represents one animal, *n* = 5–10/group.

**Figure 2.**
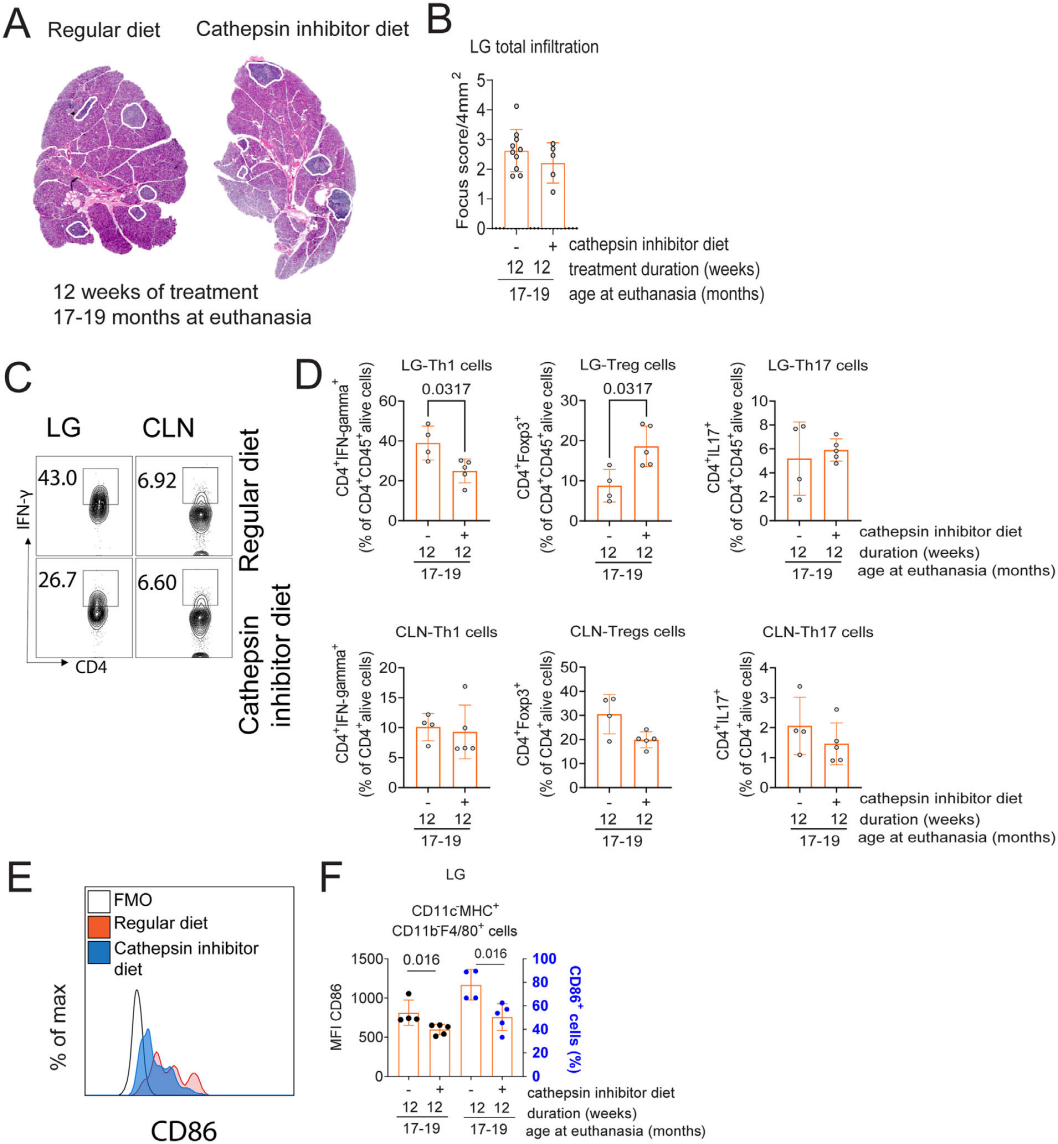
Cathepsin S inhibition diet for 12 weeks decreases CD4^+^IFN-γ^+^ cells in lacrimal glands in 17- to 19-month-old mice. (**A**) Representative whole histologic scans of aged B6 lacrimal glands with and without cathepsin S inhibition diet stained with hematoxylin and eosin. Areas of infiltration are demarcated in *white*. (**B**) Total lacrimal gland infiltration measured as focus score. Each *dot* represents one animal. Mann–Whitney *U* test, *n* = 5–10/group. (**C**) Representative flow cytometry dot plots of lacrimal gland suspensions stained with CD4 and IFN-γ. Cells were gated from CD4^+^CD45^+^ live cells. (**D**) Cumulative data of CD4^+^IFN-γ^+^ (Th1), CD4^+^IL-17^+^(Th17), and Tregs (CD4^+^ Foxp3^+^) in lacrimal glands and ocular-draining cervical lymph nodes, *n* = 4–5/group; Kruskal–Wallis comparison followed by Dunn's comparison test. (**E**, **F**) Cathepsin inhibitor diet for 12 weeks in aged mice decreases the frequency of MHC II^+^CD11b^+^F4/80^+^ cells and CD86 expression in lacrimal glands (LGs). (**E**) Representative histograms showing CD86 intensity in MHC II^+^CD11b^+^F4/80^+^ cells. (**F**) Bar graphs show cumulative data of frequency and median fluorescence intensity (MFI) of CD86, *n* = 4–5/group; Kruskal–Wallis comparison followed by Dunn's comparison test. CLN, cervical lymph node; FMO, fluorescence minus 1.

We previously reported that aged mice have increased cathepsin S activity in tears and *Ctss*^−/−^ mice have diminished age-related corneal barrier disruption and do not lose goblet cells with age.^33^ In this study, we subjected mice aged 15.5 to 17 months to a cathepsin S inhibition diet to investigate if cathepsin S blockade could be used to revert the age-related dry eye phenotype in mice.^10^^,^^44^ Mice show signs of dry eye disease as early as 12 months.^44^ Since our regimen was not meant to be preventive, we chose to evaluate aged mice that already have dry eye. Two different cohorts of mice were treated for 12 consecutive weeks, and the combined data from the two experiments are shown in Figures 1 and 2. Mice were weighed weekly, and no changes in body mass were observed after 12 weeks of diet (Supplementary Fig. S1). Aged mice on a cathepsin S inhibition diet had greater conjunctival goblet cell density and corneal mechanosensitivity than those receiving normal chow (Figs. 1A–C). These results show that a cathepsin S inhibition diet ameliorates the age-associated decrease in conjunctival goblet cell density and corneal mechanosensitivity.^40^^,^^44^^,^^45^ These results support the notion that cathepsin S inhibition improves the aged ocular surface phenotype and agree with our publication showing that aged *Ctss*^−/−^ mice are resistant to age-related dry eye disease.^33^

### Effects of Cathepsin S Inhibition in Immune Infiltration in the Aged Lacrimal Glands

Cathepsin S inhibition in autoimmune mice decreases immune infiltration in their salivary and lacrimal glands.^18^^,^^46^ As cathepsin S inhibition could also target aging-induced changes in the lacrimal gland, we quantified immune infiltration. Histopathology evaluation of aged (17- to 19-month-old) lacrimal glands 12 weeks posttreatment showed no difference in focus score/4 mm^2^ (a measurement of total lacrimal gland infiltration) (Figs. 2A, 2B).

We then characterized and quantified the immune cells infiltrating the lacrimal glands using flow cytometry (Figs. 2C, 2D). Compared to the standard diet, mice subjected to cathepsin S inhibition showed a decrease in pathogenic Th1 CD4^+^IFN-γ^+^ (49.6% ± 9% vs. 35% ± 7.5%) and an increase in regulatory CD4^+^Foxp3^+^ (9.2% ± 4% vs. 19.4% ± 5%) cells in the lacrimal glands (*n* = 5/group, *P* < 0.05 or lower). A similar non–statistically significant trend was observed in ocular draining nodes. There was no difference in the Th17 infiltration (Figs. 2C, 2D). After treatment, we also investigated the composition and activation of antigen-presenting cells in the lacrimal glands. While there was no change in the frequency of antigen-presenting cells (CD45^+^MHC II^+^ cells), mice subjected to a cathepsin S inhibitor diet had a decrease in frequency and activation (CD86 expression) in macrophages (CD45^+^CD11c^−^MHC II^+^CD11b^+^F4/80^+^ cells) in the lacrimal glands (Figs. 2E, 2F).

**Figure 3.**
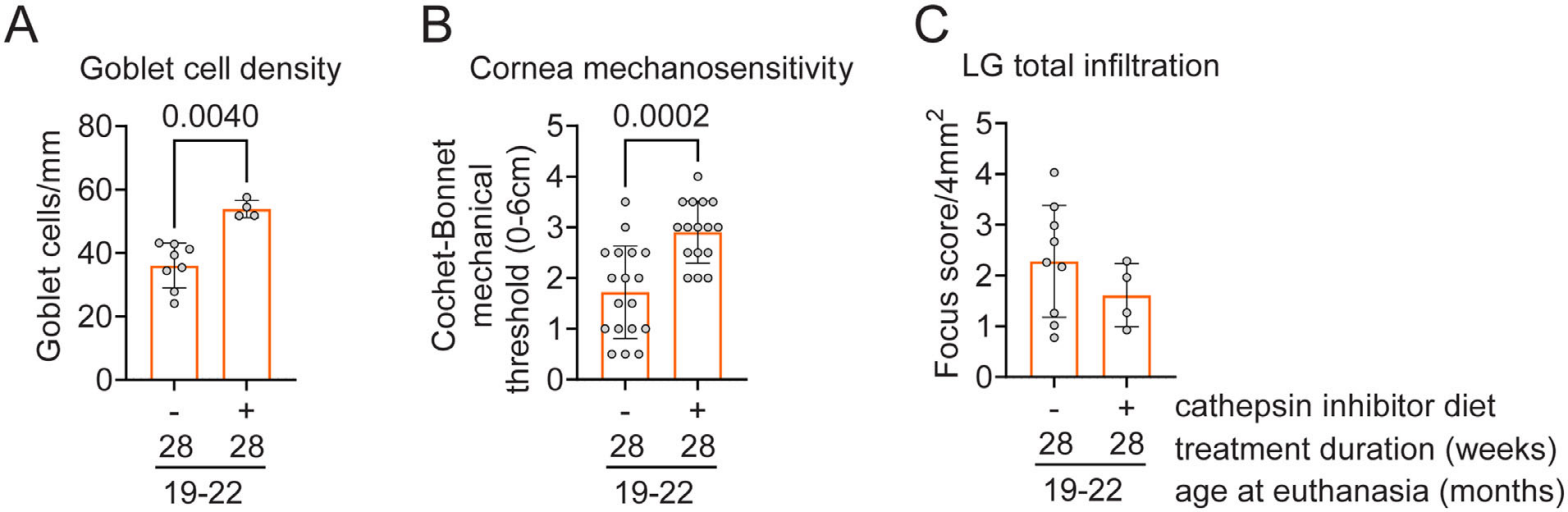
Cathepsin S inhibition diet for 28 weeks improves goblet cell density and cornea mechanosensitivity in 19- to 22-month-old mice. (**A**) Cumulative data of conjunctival goblet cell density. Mann–Whitney *U* test; each *dot* represents one animal, *n* = 4–8/group. (**B**) Cornea sensitivity was assessed using the Cochet–Bonnet aesthesiometer. Mann–Whitney *U* test; each *dot* represents one animal, *n* = 16–17/group. (**C**) Total lacrimal gland infiltration measured as focus score. Mann–Whitney *U* test. Each *dot* represents one animal, *n* = 4–8/group.

We repeated the experiment in the third cohort of mice for 28 weeks to rule out that treatment with a cathepsin S inhibitor diet for 12 weeks was insufficient to see an effect in the lacrimal gland infiltrates. In this experiment, 15.5- to 17-month-old mice received normal chow or a cathepsin S inhibition diet for 28 weeks. Mice were weighed weekly. Mice receiving a cathepsin S inhibition diet for 28 consecutive weeks gained 20% more body mass than mice receiving normal chow (Supplementary Fig. S1C). We repeated the corneal esthesiometry and conjunctival goblet cell density evaluation. Both measurements showed a significant improvement in the treated animals compared to the placebo diet (Figs. 3A, 3B), confirming our results with the 12-week treatment regimen. Next, we investigated focus scores in lacrimal gland histologic sections. Like the 12-week treatment regimen, a longer cathepsin S inhibition diet did not improve the total lacrimal gland infiltration.

These results indicate that short- and long-term treatment with cathepsin S inhibitor benefits the cornea and conjunctiva by preserving cornea sensitivity and conjunctival goblet cell density, two cardinal signs of aging in the eye. However, a cathepsin S inhibitor diet does not significantly affect ectopic lymphoid structures in the aged lacrimal gland.

### Aged *Ctss*^−/−^ Mice Are Resistant to the Age-Related Dry Eye Phenotype

**Figure 4.**
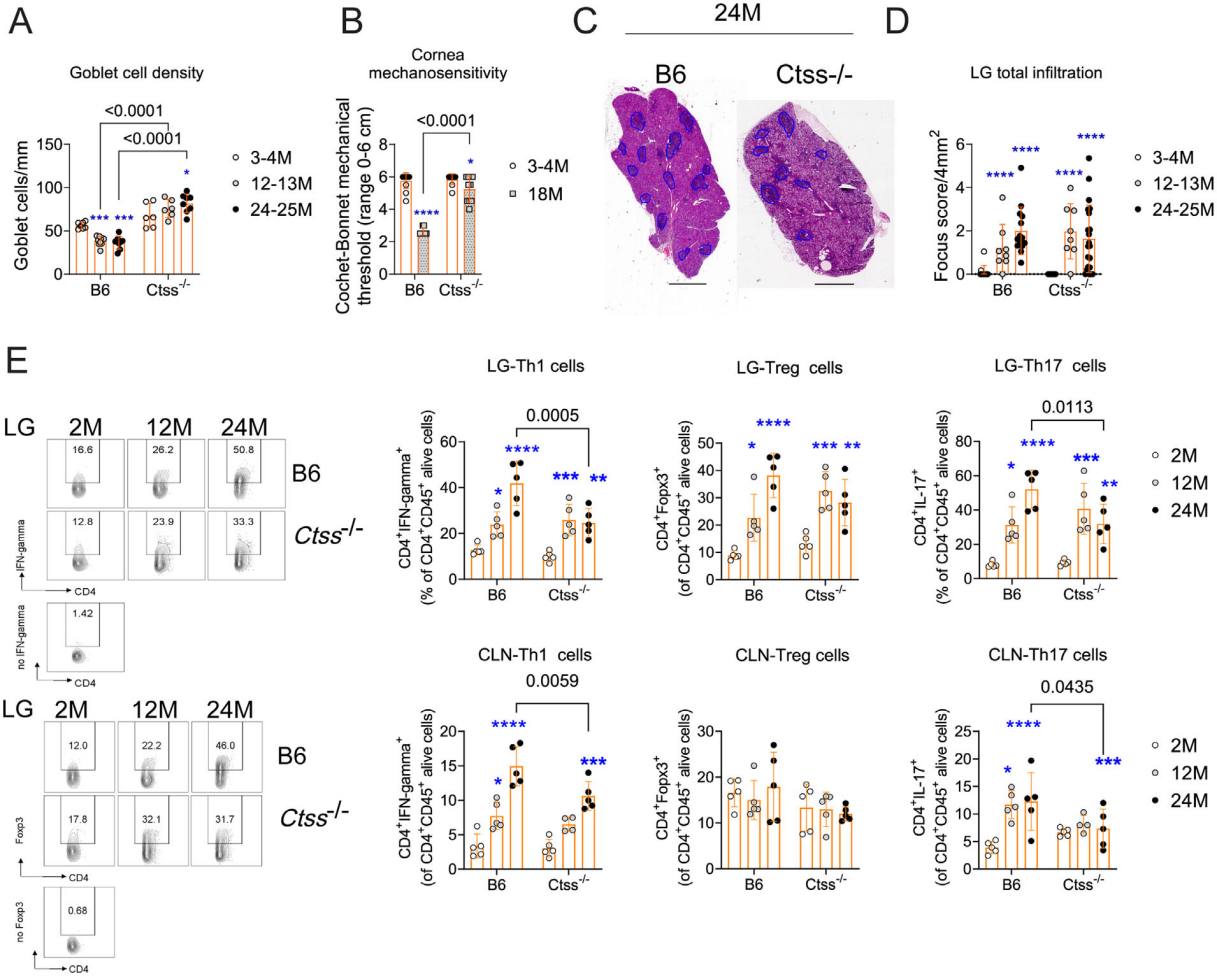
Cathepsin S genetic deletion preserves goblet cell density and cornea mechanosensitivity in aged mice while decreasing the Th1 infiltration in aged lacrimal glands. B6 mice of different ages were compared to age-matched *Ctss^−^^/^^−^* mice. Both strains of mice received standard chow. (**A**) Cumulative data of conjunctival goblet cell density. Kruskal–Wallis with Dunn's multiple comparison test. Each *dot* represents one animal, *n* = 4–8/group. Adapted from a previous publication.^33^ (**B**) Cornea sensitivity was assessed using the Cochet–Bonnet aesthesiometer. Mann–Whitney *U* test; each *dot* represents one animal, *n* = 5–8/group. (**C**) Representative whole histologic scans of aged B6 and *Ctss^−^^/^^−^* lacrimal glands stained with hematoxylin and eosin. Areas of infiltration are demarcated in *dark blue* (**C**). (**D**) Total lacrimal gland infiltration measured as focus score. Kruskal–Wallis with Dunn's multiple comparison test. *P* value as shown. *Scale bar*: 1000 µm. (**E**) Representative dot plots of lacrimal gland suspensions stained with CD4^+^IFN-γ^+^ or CD4^+^Foxp3^+^ cells. FMO, fluorescence minus 1. Cumulative data on the right showing the frequency of Th1, Tregs, or Th17 gated on CD4^+^CD45^+^ alive cells. *Asterisks* indicate intrastrain comparison of aged versus young lacrimal glands. *P < 0.05. **P < 0.01. ***P < 0.001. ****P < 0.0001. Fully written *P* values show interstrain comparisons. 3–4M, 12–13M, 24–25M = 3–4, 12–13, and 24–25 months, respectively. CLN, cervical lymph node.

Since diet-based inhibition might not completely abrogate cathepsin S activity due to pharmacologic limitations, we validated the previously described findings in aged *Ctss*^−/−^ mice. As previously shown, aged *Ctss*^−/−^ mice did not display age-related goblet cell loss (Fig. 4A).^33^ Moreover, aged *Ctss*^−/−^ mice did not display age-related corneal sensitivity loss (Fig. 4B) but had comparable total lacrimal gland infiltration to aged wild-type mice (Figs. 4C, 4D). We then characterized these immune infiltrates with flow cytometry based on several markers. While both strains have an age-related increase in Th1, Th17, and Treg cells (Fig. 4E) in their lacrimal glands, the frequency of Th1 and Th17 is smaller in the 24-month-old *Ctss*^−/−^ mice (Fig. 4E), in agreement with our previous publication showing that aged lacrimal glands have infiltrating T effectors, Tregs, and B cells.^10^^,^^43^ Similar results were present in the ocular draining cervical lymph nodes (Fig. 4E). These results confirm that cathepsin S is critical for ocular health since it preserves corneal nerve sensitivity and goblet cell density. It is also critical for preventing Th1 and Th17 infiltration in aged lacrimal glands.

### Cathepsin S Controls the T-Cell Landscape in Aged Lacrimal Glands

**Figure 5.**
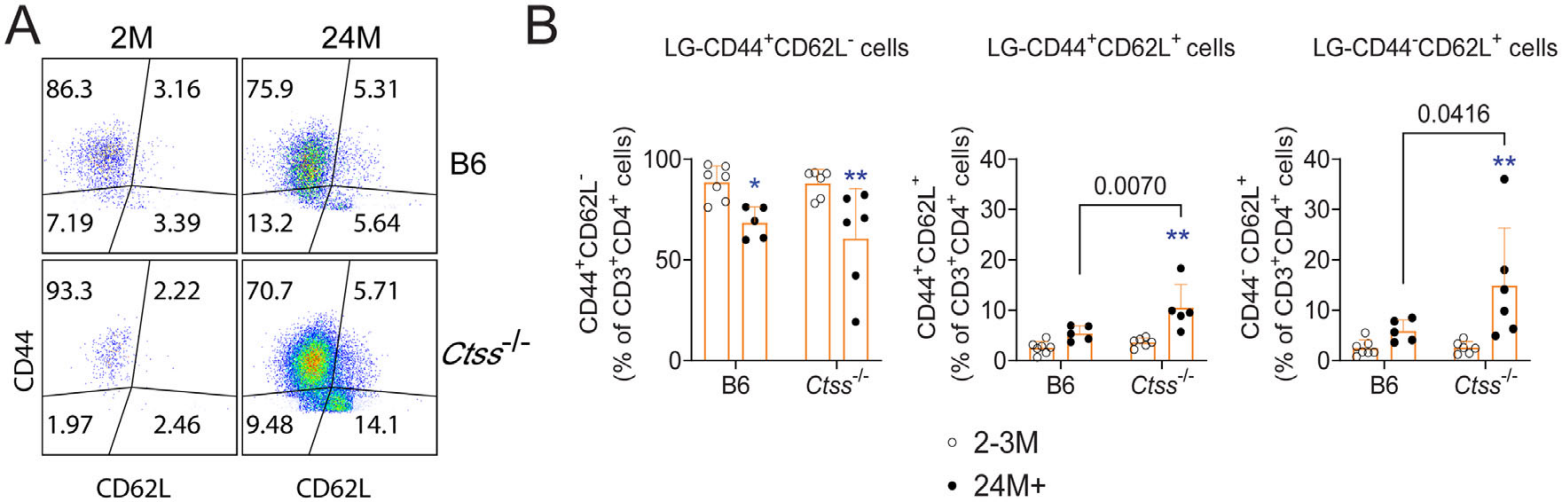
Increased T central memory (TCM) during aging in *Ctss*^−/−^ lacrimal glands. (**A**) Representative dot plots of lacrimal gland suspensions from young and aged lacrimal glands from C57BL/6 (B6) and *Ctss^−^^/^^−^* mice stained with CD44 and CD62L antibodies. (**B**) Cumulative data of frequency of CD44^+^CD62L^−^ (T effector memory), CD44^+^CD62L^+^ (T central memory), and CD44^−^CD62L^+^ (naive T cells) are shown in the graphs. Mean ± SD; each *dot* represents one lacrimal gland from one animal, *n* = 6–8/group. Kruskal–Wallis with Dunn's multiple comparison test. 2M, 24M+ = 2, 24 months (or older), respectively. *Asterisks* indicate intrastrain comparison of aged versus young lacrimal glands. **P < 0.01. Fully written *P* values show interstrain comparisons.

**Figure 6.**
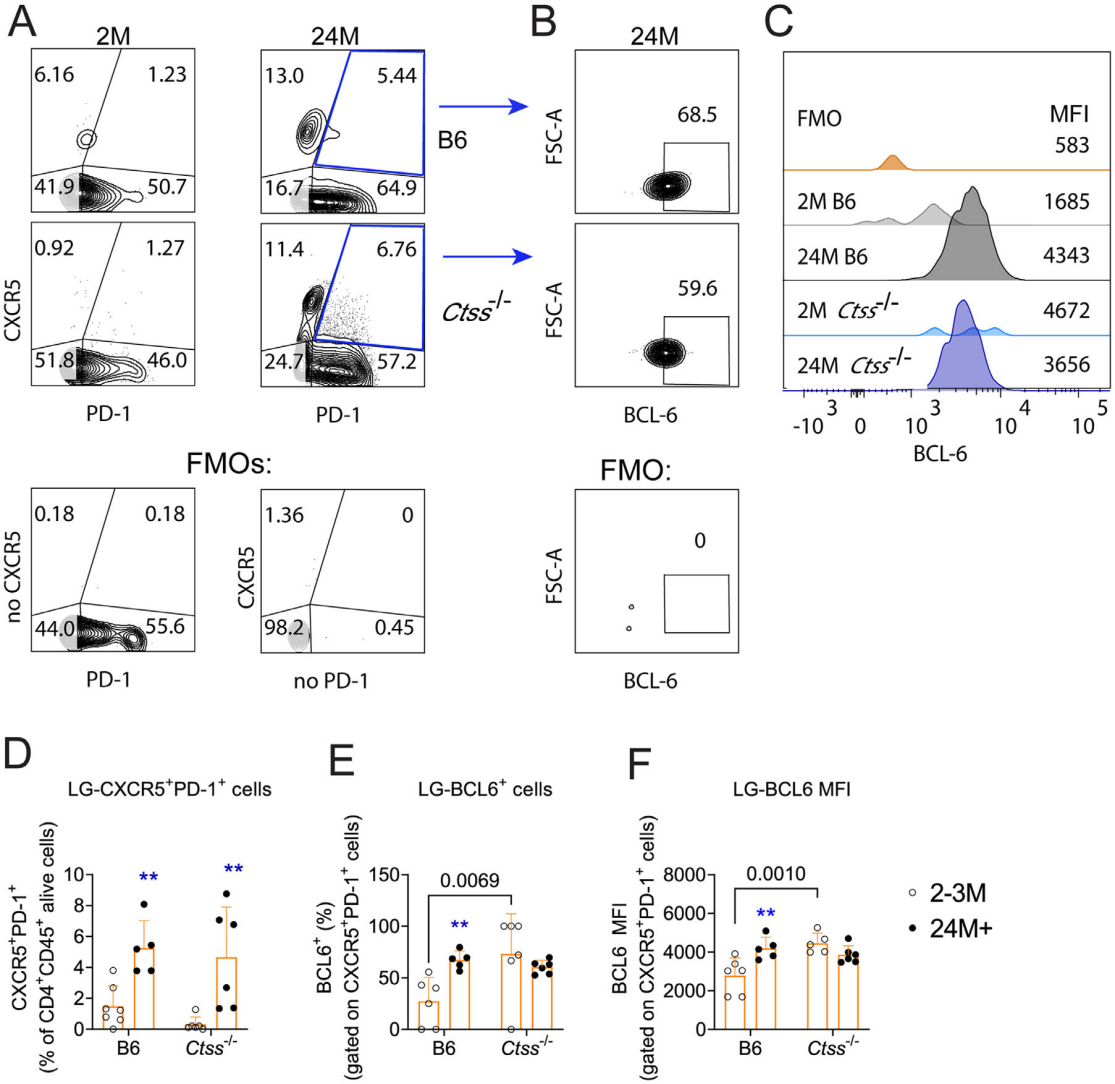
Cathepsin S controls the size of Tfh cells. (**A**) Representative gating scheme of lacrimal gland suspensions from young and aged lacrimal glands from C57BL/6 and *Ctss^−^^/^^−^* mice stained with CXCR5 and PD-1 antibodies. Cells were gated on CD4^+^CD45^+^ live cells. (**B**) Representative histograms of BCL-6 MFI in CXCR5^+^PD-1^+^ cells. (**C**) Cumulative data showing the frequency of CXCR5^+^PD-1^+^ cells. (**D**) Cumulative data of BCL-6 frequency among CXCR5^+^PD-1^+^ cells. (**E**) BCL-6 MFI of CXCR5^+^PD-1^+^ cells. Mean ± SD; each *dot* represents one lacrimal gland from one animal. Kruskal–Wallis with Dunn's multiple comparison test, *n* = 5–8. 2–3M = 2–3 months; 24M+ = 24 months or older. *Asterisks* indicate intrastrain comparison of aged versus young lacrimal glands. **P < 0.01. Fully written *P* values show interstrain comparisons.

Since we observed that aged *Ctss*^−/−^ mice had diminished Th1 and Th17 infiltration into the lacrimal glands, we performed additional flow cytometry studies investigating the frequency of naive, T central memory, and T effector memory cells in the lacrimal glands using CD44 and CD62L antibodies (Fig. 5A). The lacrimal glands of both aged *Ctss*^−/−^ and B6 mice had a decrease in the proportion of effector memory T cells (CD44^+^CD62L^−^) and an increase in the proportion of central memory and naive T cells (Fig. 5B, only significant in the aged *Ctss*^−/−^ mice). Interestingly, the proportion of central memory (CD44^+^CD62L^+^) and naive (CD44^−^CD62L^+^ cells) T cells was greater in aged *Ctss*^−/−^ than in aged B6 mice (Fig. 5B). We also investigated T follicular helper (Tfh) cell frequency using CXCR5, PD-1, and BCL-6 antibodies (Figs. 6A, 6B). In the Tfh compartment, there was an equivalent age-related increase in CD4^+^CXCR5^+^PD-1^+^ cells in both strains. Young B6 lacrimal glands had greater frequency and mean fluorescence intensity of the Tfh-specific transcription factor BCL-6 than young *Ctss*^−/−^ lacrimal glands (Figs. 6C–E). These results indicate that cathepsin S controls the pool of the different CD4^+^ T-cell compartments within the ectopic lymphoid structures that develop in the lacrimal glands during aging.

### B-Cell Infiltration Differs in the Aged *Ctss*^−/−^ Mice Compared to Wild-Type Mice

**Figure 7.**
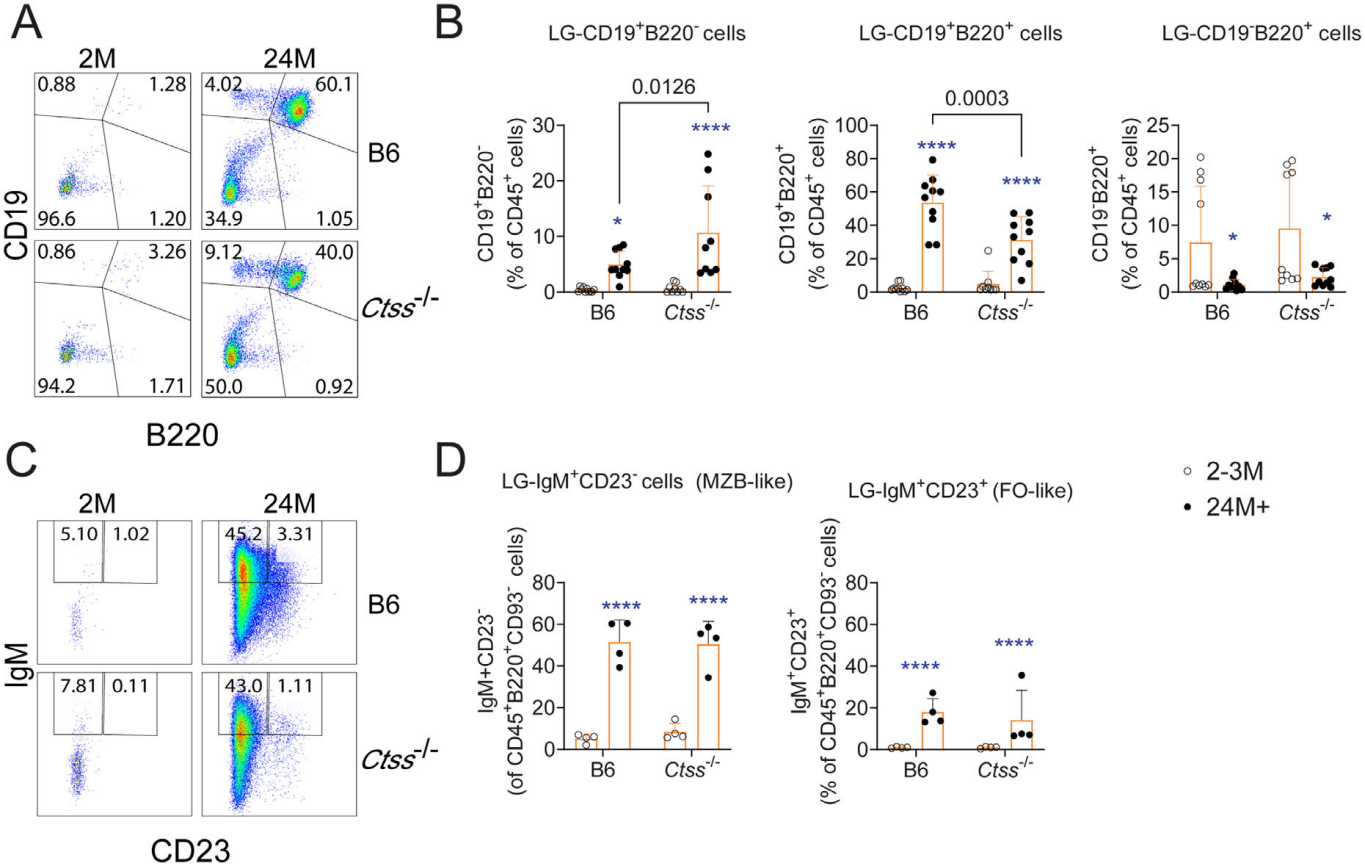
Cathepsin S controls the influx of subtypes of B cells into the aged lacrimal glands. (**A**, **B**) Representative dot plots of lacrimal gland suspensions from young and aged lacrimal glands from C57BL/6 and *Ctss*^−/−^ mice stained with CD19 and B220 antibodies (**A**) and cumulative data (**B**). Cells were gated on CD45^+^ live cells. Mean ± SD; each *dot* represents one lacrimal gland from one animal. Kruskal–Wallis with Dunn's multiple comparison test, *n* = 8–10/group. (**C**, **D**) Representative dot plots of lacrimal gland suspensions from young and aged lacrimal glands from C57BL/6 and *Ctss*^−/−^ mice stained with IgM and CD23 antibodies (**C**) and cumulative data (**D**). Cells were gated on CD45^+^B220^+^CD93^−^ cells. Mean ± SD; each *dot* represents one lacrimal gland from one animal. Kruskal–Wallis with Dunn's multiple comparison test, *n* = 4/group. 2–3M = 2–3 months; 24M+ = 24 months or older. *Asterisks* indicate intrastrain comparison of aged versus young lacrimal glands. *P < 0.05. ****P < 0.0001. Fully written *P* values show interstrain comparisons.

Most immune cells within aged lacrimal glands are B cells.^10^ B-cell expansion within these ectopic lymphoid structures relies on T-cell–derived signals elicited by B-cell–driven antigenic presentation. Cathepsin S also participates in germinal center development.^14^ Thus, we quantified the impact of *Ctss* deletion on age-associated lacrimal gland B-cell infiltrates using CD19 and B220 staining and flow cytometry analysis (Figs. 7A, 7B). We observed an increase in CD19^+^B220^−^ cells in both aged B6 and *Ctss*^−/−^ mice, but the increase was higher in *Ctss*^−/−^ than in B6 mice. On the other hand, mature CD19^+^B220^+^ cells also increased in both strains but was higher in the B6 lacrimal glands (Fig. 7B). We also further characterized the B220^+^ population into marginal zone–like cells (IgM^+^CD23^−^) or follicular-like B cells (IgM^+^CD23^+^) after gating on B220^+^CD93^−^ cells (Figs. 7C, 7D). We recently showed that aged lacrimal glands have an increase in marginal zone–like B cells.^10^ There was an age-related increase in marginal zone–like and follicular-like B cells in both strains but no difference between the aged B6 and *Ctss*^−/−^ mice. Altogether, these results indicate cathepsin S represses the CD19^+^B220^−^ cell compartment size while promoting the expansion of CD19^+^B220^+^ cells.

## Discussion

Cathepsin S levels increase in tears and lacrimal glands with aging. As augmented activity of this protease is linked with autoimmunity, its inhibition represents a potential therapeutic target for age-associated dry eye. Here we show that oral delivery of the cathepsin S inhibitor RO5461111 through specially formulated chow effectively reduces the impact of age-associated dry eye on the ocular surface of mice. These positive ocular effects are accompanied by changes in immune infiltration in the ocular draining lymph nodes and lacrimal glands. Aged *Ctss*^−/−^ mice exhibit a comparable phenotype, confirming that these findings result from the on-target effects of the cathepsin S inhibitor. Altogether, our results demonstrate the feasibility of oral administration of a cathepsin S inhibitor and its potential usefulness in ameliorating age-associated dry eye while shedding light on the underlying therapeutic mechanisms.

The combined interpretation of the data on the T- and B-cell compartments in the lacrimal glands and ocular draining lymph nodes allows us to put forth a mechanism of action in the context of age-associated dry eye. Aging impacts T-cell homeostasis through different pathways, resulting in loss of tolerance and increased autoimmunity.^1^^,^^3^^,^^47^ Since cathepsin S activity favors antigenic presentation of self-derived peptides,^14^ we believe that prolonged diet-based cathepsin S blockade interferes with the aberrant development of autoreactive T cells during aging. We have previously shown that the aging process is accompanied by the appearance of ectopic lymphoid structures with appreciable germinal centers in the lacrimal gland.^10^ In line with this, we report herein that diet-based cathepsin S inhibition modifies the age-associated immune infiltration of the lacrimal gland in terms of quality but not in quantity.

In Sjögren syndrome, a disease with lacrimal and salivary gland infiltrates, the initial events in ectopic lymphoid tissue development (i.e., lymphoid neogenesis) are related to local tissue inflammation, which ensues in response to intrinsic lacrimal gland dysfunction and acinar inflammation.^48^ Recently, a study showed that tissue-specific knockout of the autophagy pathway in acinar and ductal cells of salivary glands is sufficient to induce gland dysfunction before immune infiltration.^49^ Comparable studies on age-related lacrimal gland deficiency are lacking, but consistent with our hypothesis that loss of cell homeostasis such as in inflammation, *Tnf^−/−^* mice experience less age-related infiltration and decreased number of ectopic lymphoid structures.^50^^,^^51^ Contrasting with the initial events of lymphoid neogenesis, its progression requires antigen presentation, which in this context probably involves self-antigens. This process ensures adequate T follicular and B-cell cooperation and B-cell expansion. We propose that diet-based cathepsin S inhibition prevents autoantigen presentation in this scenario, as it has been demonstrated for young autoimmune-prone mice.^18^^,^^46^ In line with this, we observed fewer activated macrophages (professional antigen-presenting cells), fewer proinflammatory Th1 CD4^+^ T cells, and more regulatory CD4^+^ T cells, in agreement with our previous publications that these cells are important players in age-related dry eye.^43^^,^^52^ Aged cathepsin S–deficient mice replicated these findings, confirming that they were due to the specific blockade of cathepsin S activity by the inhibitor diet. Supporting our hypothesis, aged *Ctss*^−/−^ mice had a reduced proportion of effector memory T cells and a higher proportion of naive and central memory T cells within the lacrimal gland infiltrates. Moreover, these mice harbored fewer T follicular cells in the lacrimal glands as assessed by the expression of the signature transcription factor *Bcl-6*. These cells are required to drive T-cell–dependent B-cell responses.^53^^,^^54^ Like the antigen-presenting cells that activate and induce T follicular cells, B cells also rely on cathepsin S for MHC II processing and thus obtain cooperative signaling from cognate antigen-specific T follicular cells.^55^ Therefore, cathepsin S is also essential for the expansion and proliferation of the corresponding B cells in physiologic humoral immune responses,^13^ but cathepsin S inhibition does not alter T-cell–independent responses. Our findings on the landscape of the lacrimal gland infiltrates of aged *Ctss*^−/−^ mice are consistent with this framework: we observed an increased proportion of CD19^+^B220^−^ B cells, which do not rely on T cells for antibody production, and a reduced proportion of CD19^+^B220^+^ B cells, which depend on T follicular cells for expansion.^56^ Thus, the changes in both T- and B-cell compartments within the lacrimal gland ectopic lymphoid tissue of aged cathepsin S-deficient mice are likely linked by the reduction in local autoantigen presentation. However, because the antigen in dry eye remains elusive, further studies are necessary to investigate this.

By contrast, the beneficial effects of the cathepsin S inhibitor-containing diet on the ocular surface of aged mice are probably due to the blockade of extracellular cathepsin S activity. We have shown that aged C57BL/6J mice have increased cathepsin S activity levels in tears and lacrimal gland lysates.^33^ The source of increased cathepsin S levels in aged tears is most likely dysregulated exocytosis of endolysosomal vesicles in the lacrimal gland acinar cells, as it has been demonstrated to occur in a murine Sjögren syndrome model.^57^ Cathepsin S exocytosis is also differentially modulated by sympathetic and parasympathetic autonomic stimuli.^58^ Intriguingly, the increase in sympathetic activity that occurs with aging^59^ is associated with increased cathepsin S secretion through an alternative pathway in lacrimal gland acinar cells.^58^ Regarding its pathogenic action, cathepsin S disrupts intercellular tight junctions in cultured corneal epithelial cells, and topical ocular administration to *Ctss*^−/−^ mice breaks down the corneal epithelial barrier, thus replicating the phenotype.^33^ In addition, cathepsin S elicits extracellular matrix remodeling in other tissues^60^^,^^61^ and can degrade the lubricating proteoglycans of the ocular surface.^62^ Contrasting the knowledge of its pathogenic effects on the corneal epithelium, the impact of cathepsin S on corneal nerves and conjunctival goblet cells is less understood. IFN-γ deficiency protects mice from age-related goblet cell loss.^40^ As both corneal nerves and goblet cells are negatively affected by Th1 CD4^+^ T cells,^41^^,^^63^^–^^66^ it is tempting to speculate that cathepsin S inhibition during aging protects corneal nerves and goblet cells by reducing Th1 immune responses on the ocular surface. Supporting this idea, we observed fewer IFN-γ–producing CD4^+^ T cells in the lacrimal glands and a comparable trend in the eye-draining lymph nodes. However, more work is warranted to prove or discard this hypothetical mechanism of action.

Our research provides preclinical evidence that an oral approach to cathepsin S inhibition could be therapeutic in age-related dry eye. Comparable strategies have shown preclinical efficacy in Sjögren syndrome^19^^,^^67^ using a different small molecule (RO5459072). By contrast, a recent phase II study that evaluated a 12-week-long daily oral intake of RO5459072 cathepsin S inhibitor in patients with Sjögren syndrome did not detect a clinical benefit.^68^ Nonetheless, these findings do not necessarily rule out the therapeutical potential of cathepsin S blockade in this disease context for two reasons. First, assessing improvement in Sjögren syndrome is challenging as it relies mainly on subjective scoring that compounds into a disease activity index.^69^ Therefore, the study documented a high individual variation.^68^ Second, the optimal duration for a treatment to show improvement in a chronic condition such as Sjögren syndrome is not known beforehand, especially with a therapeutic intervention that targets long-standing pathogenic processes (i.e., autoimmune responses). Both concerns also apply to age-related dry eye. In this regard, herein we show that 12-week and 28-week-long treatments with a cathepsin S inhibitor improved age-related dry eye parameters, a strength of our findings. Thus, dietary blockade of cathepsin S represents a potential therapeutic intervention in age-associated dry eye. Further studies are necessary to investigate if topical eye drops would be as equally beneficial as dietary intake.

## Supplementary Material

Supplement 1
