# Supplementary material for: Effects of Cathepsin S Inhibition in the Age-Related Dry Eye Phenotype
Source: Invest Ophthalmol Vis Sci. 2023 Aug 4;64(11):7. doi: 10.1167/iovs.64.11.7 (PMC10414132; doi:10.1167/iovs.64.11.7)
Supplement: Supplement 1 [file iovs-64-11-7_s001.pdf]

Effects of Cathepsin S Inhibition in the Age-Related Dry Eye Phenotype

Jeremias G. Galletti, Kaitlin K. Scholand, Claudia M. Trujillo-Vargas, Wolfgang Haap, Tiago Santos-Ferreira, Christoph Ulmer, Zhiyuan Yu, Cintia S. de Paiva

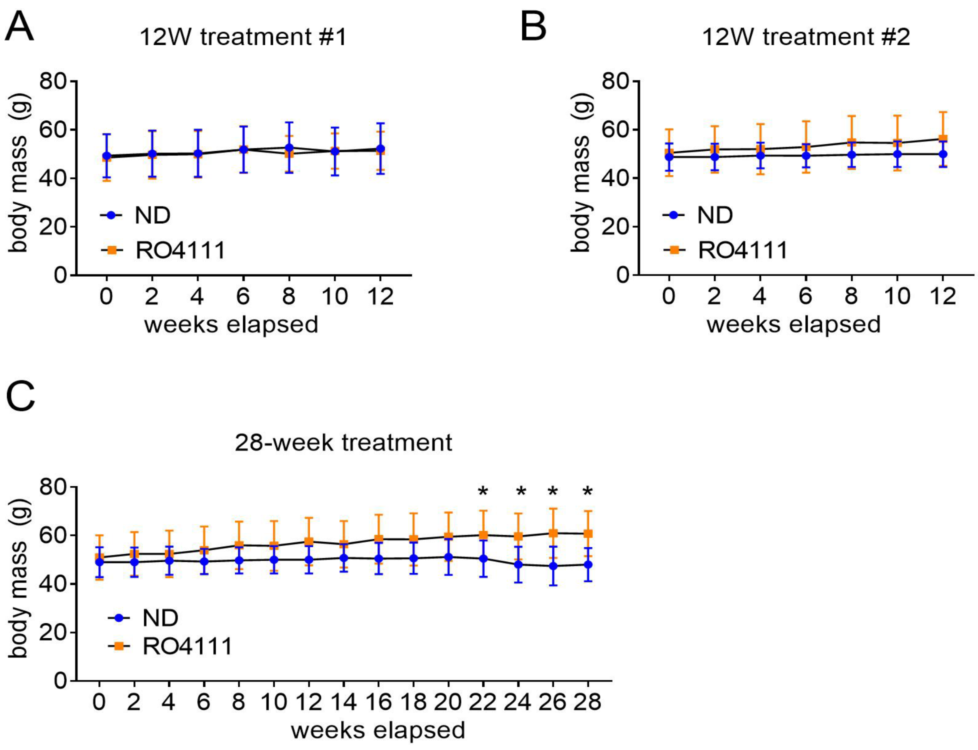

Supplemental Figure 1: Body mass measurements in the three different cohorts.

15.5-17-month-old female C57BL/6 mice received a medicated chow containing RO5464111 or normal diet (ND) for 12 (A, B) or 28 consecutive weeks (C). 2-way ANOVA followed by Sidak's multiple comparison test. * $P < 0.05$
